# MicroRNA Isoforms Contribution to Melanoma Pathogenesis

**DOI:** 10.3390/ncrna7040063

**Published:** 2021-09-27

**Authors:** Elisabetta Broseghini, Emi Dika, Eric Londin, Manuela Ferracin

**Affiliations:** 1 Department of Experimental, Diagnostic and Specialty Medicine (DIMES), University of Bologna, 40126 Bologna, Italy; elisabett.broseghin2@unibo.it (E.B.); emi.dika3@unibo.it (E.D.); 2 Dermatology Unit, IRCCS Azienda Ospedaliero-Universitaria di Bologna, 40138 Bologna, Italy; 3 Computational Medicine Center, Sidney Kimmel Medical College, Thomas Jefferson University, Philadelphia, PA 19107, USA; Eric.Londin@jefferson.edu

**Keywords:** melanoma, isomiR, next generation sequencing, TCGA

## Abstract

Cutaneous melanoma (CM) is the most lethal tumor among skin cancers, and its incidence is constantly increasing. A deeper understanding of the molecular processes guiding melanoma pathogenesis could improve diagnosis, treatment and prognosis. MicroRNAs play a key role in melanoma biology. Recently, next generation sequencing (NGS) experiments, designed to assess small-RNA expression, revealed the existence of microRNA variants with different length and sequence. These microRNA isoforms are known as isomiRs and provide an additional layer to the complex non-coding RNA world. Here, we collected data from NGS experiments to provide a comprehensive characterization of miRNA and isomiR dysregulation in benign nevi (BN) and early-stage melanomas. We observed that melanoma and BN express different and specific isomiRs and have a different isomiR abundance distribution. Moreover, isomiRs from the same microRNA can have opposite expression trends between groups. Using The Cancer Genome Atlas (TCGA) dataset of skin cancers, we analyzed isomiR expression in primary melanoma and melanoma metastasis and tested their association with NF1, BRAF and NRAS mutations. IsomiRs differentially expressed were identified and catalogued with reference to the canonical form. The reported non-random dysregulation of specific isomiRs contributes to the understanding of the complex melanoma pathogenesis and serves as the basis for further functional studies.

## 1. Introduction

Malignant cutaneous melanoma (CM) results from transformation of pigments-producing cells known as melanocytes. Worldwide incidence of melanoma has progressively increased over the last several decades, reaching an age-standardized rate of 3.4 per 100,000. However, in fair-skinned population regions, including Australia-New Zealand, Northern Europe and Northern America, this rate exceeds 15 per 100,000 (35.8, 17.8 and 16.1, respectively) (source IARC 2020).

Although malignant melanoma accounts only 3–5% of all skin cancers, it is the most lethal form, and determines up to 65% of the deaths [1]. The pathogenesis of melanoma is complex, and the onset of melanoma has been associated with several risk factors that include genetic, environmental, and phenotypic factors such as ultraviolet (UV) exposure, fair phototypes, multiple dysplastic nevi and a positive family/personal history of cutaneous melanoma [2].

Melanoma risk is increased in people harboring specific germline genetic alterations [3], which include mutations in Cyclin Dependent Kinase Inhibitor 2A (*CDKN2A*) gene [4,5], Cyclin Dependent Kinase 4 (*CDK4*), Telomerase Reverse Transcriptase (*TERT*), Microphthalmia-associated transcription (*MITF*), BRCA1-associated protein-1 (*BAP1*), and Protection of telomeres 1 (*POT1*) [6]. Sporadic cutaneous melanoma is characterized by one of the highest tumor mutation rates, mainly due to UV exposure [7]. The most frequently mutated genes in sporadic melanoma are B-Raf Proto-Oncogene (also known as v-Raf murine sarcoma viral oncogene homolog B, *BRAF*), neuroblastoma RAS viral oncogene homolog (*N-RAS*), Neurofibromin 1 (*NF1*), proto-oncogene receptor tyrosine kinase (*KIT*) and Phosphatase and Tensin homolog (*PTEN*) [8]. Based on the pattern of mutated genes, a genomic classification of melanoma has been established and melanomas have been classified into four subtypes: mutant *BRAF*, mutant *RAS*, mutant *NF1*, and Triple-WT (wild-type) [9].

The gain-of function mutations of BRAF and NRAS lead to constitutive activation of the signaling of the mitogen-activated protein kinase (MAPK) pathway and thus to tumor proliferation and progression [10]. On the other hand, the loss-of function of NF1 leads to hyperactivation of RAS proteins and to promotion of MAPK pathway signaling [11]. The discovery that BRAF and NRAS mutations are two of the main oncogenic drivers of melanoma proliferation and survival has resulted in important therapeutic implications and changed the management of cutaneous melanoma patients with the development of specific targeted therapies based on MAPK pathway inhibitors [12].

Important molecular factors in melanoma pathogenesis are microRNAs (miRNAs). miRNAs are small non-coding RNAs (18–22 nucleotides in length) that regulate multiple pathways with an oncogenic or tumor suppressive role [13,14,15]. Normal and pathological cells and tissues can be classified using the global miRNA expression profile [16,17].

In the past decade, the characterization of the tissue miRNA profile has taken advantage of the spreading of deep sequencing techniques to investigate the sequence and abundance of small noncoding RNAs. Most small RNA sequencing studies focused on the identification of canonical miRNAs, whose sequence is reported in the miRBase database [18]. Recently, many deep sequencing experiments in human tissues evidenced the existence of alterations in miRNA length and sequence [19], suggesting the importance of including the analysis of miRNA variants or isoforms (isomiRs) in miRNA studies. The isomiR biogenesis has not been completely clarified yet, but it seems that isomiRs could derive from alternative Drosha and or Dicer cleavage of the precursor miRNAs [20], or from post-transcriptional modifications made by nucleotidyl transferase, which adds nucleotides to the pre-miRNA or mature miRNA ends [21,22,23]. Stability and abundance can vary between the canonical miRNA and its isoforms. The proposed role of isomiRs is to reinforce the regulatory activity of canonical miRNAs [24], though there is still much to discover. Furthermore, isomiR expression can discriminate human cancers [25].

Recently, we investigated the global canonical miRNA profile of formalin-fixed paraffin-embedded (FFPE) samples of benign nevi, single and multiple primary melanomas [26]. In addition, we analyzed the smallRNA sequencing data at the isomiR level. Combining all the samples together, we identified a panel of highly expressed isomiRs, with an expression up to 10 times higher than the canonical miRNA.

This prompted us to further investigate the contribution of isomiRs in melanoma progression. Specifically, in this study we identify and classify isomiRs detectable in FFPE benign nevi and melanoma samples using proprietary small RNA sequencing data and assess isomiR expression in relation to the corresponding canonical miRNA. In addition, we compare the isomiR expression profile of benign nevus and melanoma. Finally, using publicly available smallRNA sequencing data from fresh-frozen skin cutaneous melanoma (SKCM) of The Cancer Genome Atlas (TCGA) database, we identify and compare the isomiR expression profile of primary melanoma and metastasis, and identify isomiRs whose expression is associated with NF1, BRAF and NRAS mutations.

In what follows, the term “canonical” has been used to indicate the sequence of a microRNA as reported in miRBase. On the other hand, “isomiRs”, “miRNA variants” and “miRNA isoforms” indicates all the variant forms that differentiate from the canonical miRNA. Finally, the set of canonical miRNAs and their isomiRs has been grouped and called “mature microRNAs”.

## 2. Results

### 2.1. Mature microRNA Profile and miRNA Variants Characterization in Early-Stage Melanoma Samples

**Figure 1:**
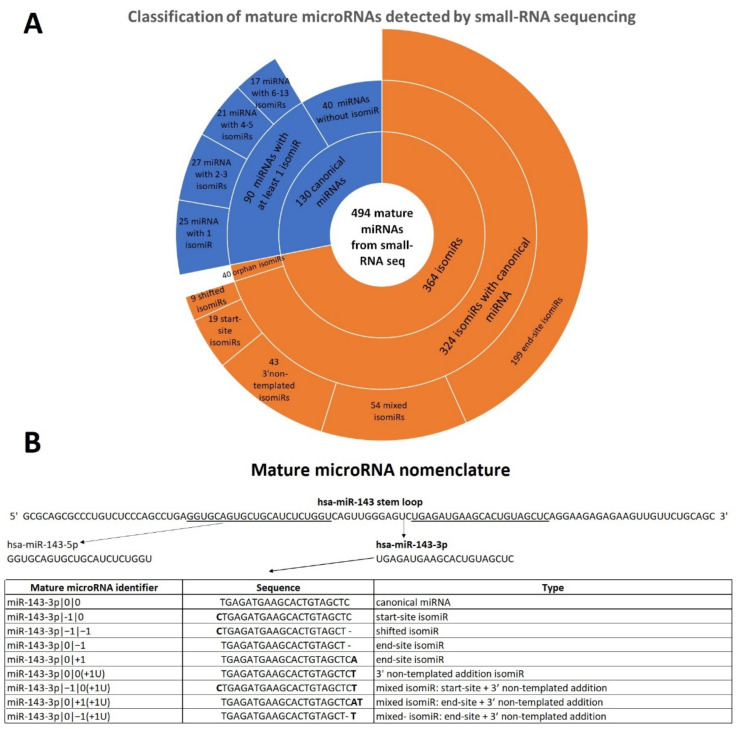
Mature microRNAs classification and nomenclature. (**A**) Classification of all mature microRNAs detected by small-RNA sequencing in 23 formalin-fixed paraffin-embedded (FFPE) samples (20 cutaneous melanoma (CM) and 3 benign nevi (BN)). A sunburst chart represents the type of mature microRNAs detected in small-RNA seq data. (**B**) Example of mature microRNA nomenclature of miR-143-3p. miR-143 stem loop originates two canonical miRNAs, namely miR-143-5p and miR-143-3p. In our analysis, nine mature microRNAs of miR-143-3p were detected. The table reports nomenclature, sequence and type of mature microRNA. Nucleotides in bold corresponds to additional nucleotides compared to the canonical sequence; “-“ indicates the deletion of a nucleotide compared to the canonical sequence.

We analyzed the global mature microRNA (canonical and isomiR) profile of 23 FFPE samples: three benign nevi (BN) and 20 early-stage primary cutaneous melanoma (CM) samples [26]. Using a dedicated bioinformatics pipeline called isoMiRmap [27], we identified 494 mature microRNAs, including 130 canonical miRNAs and 364 microRNA isoforms that passed quality trimming and filtering. Specifically, we identified 90 canonical miRNAs with at least one isomiR, for a total of 324 isomiRs with a detectable canonical form. About 28% of these 90 canonical miRNAs had only one isomiR, 30% had two or three isomiRs and the remained 42% consisted in miRNAs that had a number of isomiRs that ranged from four to thirteen (Table 1, Figure 1A). The characterization of all mature miRNAs evidenced that 40 expressed canonical miRNAs did not have any isomiR in our data. As a counterpart, canonical miRNA sequences were not detected in our dataset for about 11% of the identified isomiRs. In this case, isomiRs are called “orphan” isomiRs (Supplementary Table S1).

Since isomiR sequences differentiate from canonical miRNAs in different ways, we classified them into five categories: start-site isomiR, end-site isomiR, 3′ non-templated addition isomiR, shifted isomiR and the remaining “mixed” isomiR. The first and second groups present nucleotides addition or deletion respectively at the 5′ and at the 3′ end of the canonical sequence. In these two groups, added nucleotides “match” with the sequence of the stem loop. On the contrary, when the added nucleotides in 5′ or 3′ end are different compared to those in the stem loop sequence, we defined 5′ non-templated addition and 3′ non-templated addition isomiRs, respectively. In our dataset, we did not identify any 5′ non-templated addition isomiR. Conversely, several isomiRs present 3′ non-templated addition and most of them consist in uridylation, namely addition of one or more uracils. Shifted isomiRs are long as the canonical counterpart, however their start-sites and, consequently, their end-sites are shifted to the left (in 5′ direction) or to the right (in 3′ direction) to one or more positions. Finally, the mixed isomiR group includes isomiRs that manifest at least two of these differences compared to the canonical form. An example of isomiR classification is reported in Figure 1B.

### 2.2. End-Site isomiRs Are the Most Abundant and Expressed isomiRs in FFPE Samples

Most of the detected isomiRs belonged to the end-site class of isomiRs (61.4%), while the other four classes were less represented. In addition, looking at the mixed group, we observed a prevalence of isomiRs that present the combination of different end-site modifications (deletions or additions of “matched” nucleotides) and 3′ non-templated additions when compared to canonical miRNA (Table 1).

Looking at the normalized expression of the 364 isomiRs in the 20 FFPE early-stage melanoma samples, we observed that they can vary between 5–6 to over 2300. We selected the most expressed isomiRs in these 20 CM samples (normalized expression >20) and obtained a list of 126 isomiRs that are reported in Supplementary Table S2. We found that the majority of expressed isomiRs (about 75%) belong to the end-site group. Less frequent are the isomiRs belonging to the other groups: 9% mixed group, 9% 3′ non-template addition group, 4% start-site group and 3% shifted group.

Among the ten most expressed isomiRs we found nine end-site isomiRs (hsa-miR-10b-5p|0|−1, hsa-miR-205-5p|0|+1, hsa-miR-27b-3p|0|−1, hsa-miR-125a-5p|0|−2, hsa-miR-10a-5p|0|−1, hsa-miR-181a-5p|0|−1, hsa-miR-30d-5p|0|+2, hsa-miR-143-3p|0|−1, hsa-let-7a-5p|0|−1) and one 3′ non-template addition isomiR (hsa-miR-143-3p|0|0(+1U)).

### 2.3. Identification of isomiRs More Expressed Than the Canonical Form in Early-Stage Melanoma

To identify the most relevant isomiRs in early-stage melanoma, we focused on isomiRs that showed a higher or an equal expression level than the corresponding canonical mRNA form. For this purpose, we calculated the expression ratio between isomiRs and canonical miRNA. For this analysis, we excluded miRNAs without isomiRs and orphan isomiRs. We considered the average expression of the selected 412 mature miRNAs in 20 early-stage melanomas and calculated the expression ratio for each isomiR. We obtained a wide set of ratios: some isomiRs are expressed up to 3000 times less than the canonical miRNAs, as expected, but some isomiRs are upregulated up to 50 times more than their canonical counterparts (Supplementary Table S3).

**Figure 2:**
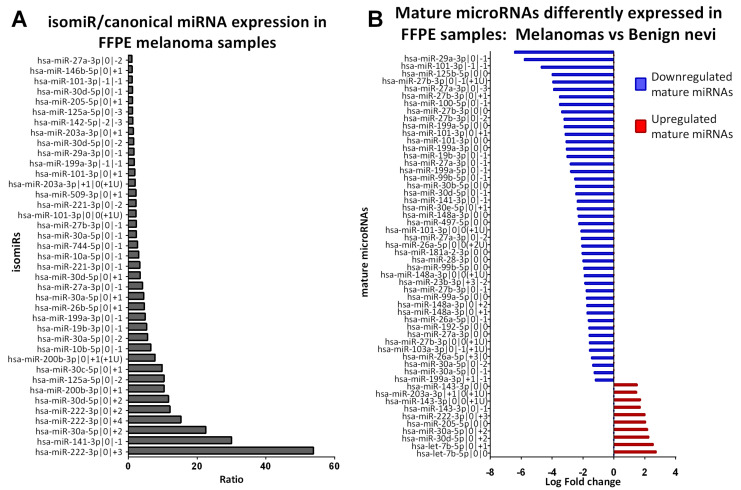
Expression of mature microRNAs in FFPE samples. (**A**) IsomiR/canonical miRNA expression in 20 FFPE melanoma samples. Bar graph representing isomiR/miRNA ratios in 20 FFPE early-stage melanoma samples greater than 1 for isomiR with an average normalized expression >20 (*n* = 39). (**B**) List of 55 mature microRNAs differently expressed in 20 FFPE early-stage melanoma compared to 3 FFPE benign nevi samples. Interleaved bar charts showing the average log fold change in CM vs. BN. We identified 10 upregulated (in red) and 45 downregulated mature microRNAs with Fold change >2 and adjusted *p* value < 0.05.

We decided to filter our results in order to identify isomiRs that could play a role in melanoma pathogenesis. We selected isomiRs with a ratio isomiR/miRNA >1 from Supplementary Table S3, obtaining a list of 39 isomiRs (Figure 2A). Some of these isomiRs belonged to the same miRNA family. Specifically, nine isomiRs belong to miR-30 family (3 tomiR-30a-5p, 3 to miR-30d-5p and 1 to miR-30c-5p); five isomiRs belong to miR-221/222 family (3 to miR-221-3p, 2 to miR-222-3p); four isomiRs belong to miR-10 family (2 to miR-125a-5p, 1 to miR-10a-5p, 1 to miR-10b-5p) three isomiRs belong to miR-101 family (all to miR-101-3p), three isomiRs belong to miR-200 family (2 to miR-200b-3p, 1 to miR-141-3p), and three isomiRs belong to miR-27 family (2 to miR-27b-3p, 1 to miR-27a-3p) (Table 2).

### 2.4. Benign Nevi and Early-Stage Melanoma Present a Different Mature microRNA Expression Profile

We compared the mature miRNA (canonical and isomiR) expression profile of 3 benign nevi and 20 early-stage primary cutaneous melanoma. We identified 55 mature miRNAs that are differentially expressed between early-stage CM and BN (adjusted *p* < 0.05, Table 3). Their fold change is represented with a horizontal histogram graph in Figure 2B. Most of them (*n* = 45) are downregulated in melanoma, while 10 are upregulated.

Among the differentially expressed mature miRNAs, we found 18 canonical miRNAs and 37 isomiRs. Among the canonical miRNAs, 10 have at least one isomiR in this list of 55 mature miRNAs ((let-7b-5p, miR-101-3p, miR-143-3p, miR-148a-3p, miR-199a-3p, miR-199a-5p, miR-27a-3p, miR-27b-3p, miR-29a-3p, miR-99b-5p), while 8 do not have any isomiR in the list (miR-125b-5p, miR-181a-2-3p, miR-192-5p, miR-205-5p, miR-28-3p, miR-30b-5p, miR-497-5p, miR-99a-5p). For 16 isomiRs the canonical miRNA is not present in the list, meaning that the isomiR is differently expressed in early-stage CM vs. BN, but not the canonical miRNA.

In most cases, variant isoforms show the same expression variation than the canonical miRNA, namely isomiR(s) and the corresponding canonical miRNA are all upregulated or downregulated in the melanoma group compared to the benign nevi group. However, we found some isomiRs of the same canonical miRNA that present opposite trends. Specifically, miR-30d-5p and miR-30a-5p have isomiRs with reverse trends: miR-30a-5p|0|+2 and miR-30d-5p|0|+2 are upregulated in early-stage melanoma, while miR-30a-5p|0|−1, miR-30a-5p|0|−2 and miR-30d-5p|0|−1 are downregulated compared to benign nevi samples. Canonical miRNAs of miR-30a-5p and miR-30d-3p are not differentially expressed between CM and BN.

Among the 37 isomiRs that are differently expressed in early-stage CM vs. BN, there are 16 miRNAs with a ratio isomiR/miRNA > 1 (Table 2). In particular, hsa-miR-101-3p|0|+1, hsa-miR-101-3p|0|0(+1U), hsa-miR-101-3p|−1|−1, hsa-miR-141-3p|0|−1, hsa-miR-19b-3p|0|−1, hsa-miR-27a-3p|0|−1, hsa-miR-27a-3p|0|−2, hsa-miR-27b-3p|0|−1, hsa-miR-29a-3p|0|−1, hsa-miR-30a-5p|0|−1, hsa-miR-30a-5p|0|−2, and hsa-miR-30d-5p|0|−1 are downregulated in early-stage CM, while hsa-miR-203a-3p|+1|0(+1U), hsa-miR-222-3p|0|+3, hsa-miR-30a-5p|0|+2, and hsa-miR-30d-5p|0|+2 are upregulated in early-stage CM (Table 2 and Table 3).

### 2.5. Classification of isomiRs with a Potentially Relevant Role in Early-Stage Melanoma

We decided to further investigate the expression of mature miRNAs in FFPE samples showing two characteristics: (i) at least one isoform is differentially expressed between early-stage CM and BN and (ii) at least one isoform is more expressed than the canonical form. We obtained a list of 10 miRNAs, which were further divided into four classes: (a) isomiRs with a similar trend in early-stage CM vs. BN and similar relative abundance; (b) isomiRs with a similar trend in early-stage CM vs. BN and different relative abundance; (c) isomiRs with opposite trend in early-stage CM vs. BN and similar relative abundance; and (d) isomiRs with opposite trend in early-stage CM vs. BN and different relative abundance.

#### 2.5.1. IsomiRs with a Similar Trend in Early-Stage CM vs. BN and Similar Relative Abundance

**Figure 3:**
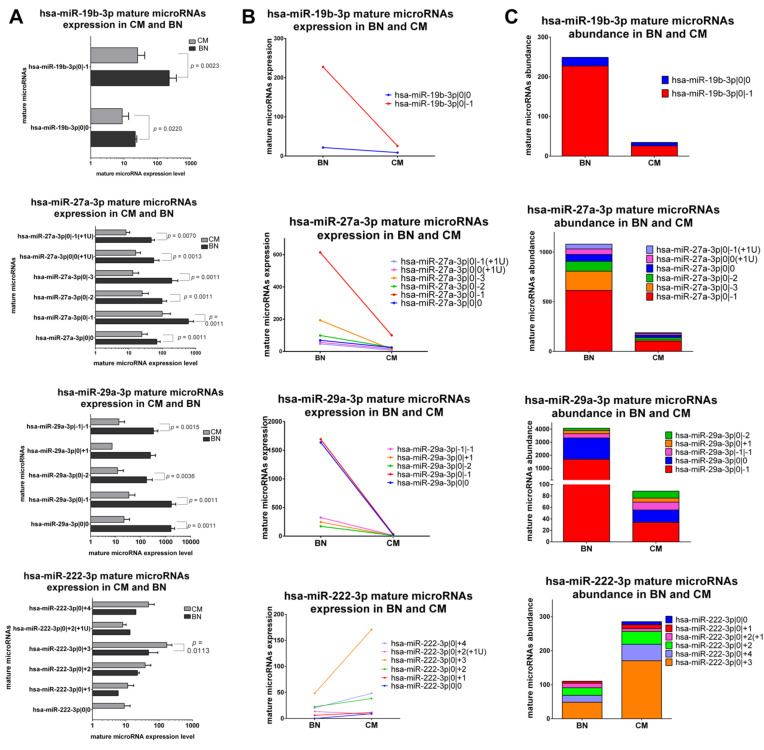
Group 1: IsomiRs with a similar trend in 20 FFPE CM vs. 3 FFPE BN and similar relative abundance. (**A**) Mature microRNA expression in BN and CM. All mature microRNAs are represented in a separated bar chart (mean ± standard deviation). A Mann-Whitney test was performed to compare the expression level in BN and CM. *P values* are shown when the difference is statistically significant. (**B**) Expression trend of mature microRNAs in BN and primary CM. Before-after blot shown the same expression trend between mature microRNAs. (**C**) Mature microRNA abundance distribution microRNAs in BN and CM. Stacked bar chart illustrates the same mature microRNA abundance distribution between BN and CM. Mature microRNAs are sorting based on their expression in BN.

The first class includes canonical miRNAs and corresponding isomiRs with the same trend of variation between early-stage CM and BN and a similar abundance distribution. miR-19b-3p, miR-27a-3p, miR-29a-3p and miR-222-3p belong to this class. The first three canonical miRNAs and their isomiRs are downregulated in CM, while mature microRNAs of miR-222-3p are upregulated in CM (Figure 3A–C). Also the relative abundance is similar in CM and BN. In fact, both in CM and BN the most expressed isomiRs are miR-19b-3p|0|−1, 27a-3p|0|−1, miR-29a-3p|0|−1 followed by the canonical miR-29a-3p and miR-222-3p|0|+3 (Figure 3).

#### 2.5.2. IsomiRs with a Similar Trend in CM vs. BN and Different Relative Abundance

**Figure 4:**
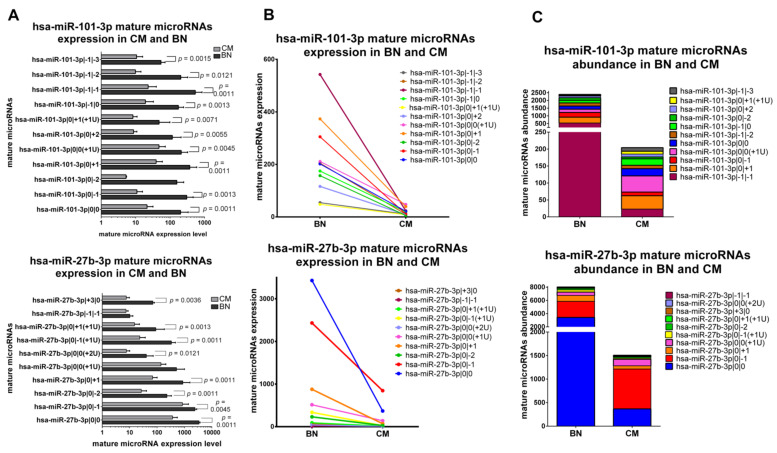
Group 2: IsomiRs with a similar trend in 20 CM vs. 3 BN and different relative abundance. (**A**) Mature microRNA in BN and CM. All mature microRNAs are represented in a separated bar chart (mean). Bar indicates standard deviation. Mann-Whitney t test was performed to compared expression level in BN and CM. *P values* are shown when the difference is statistically significant. (**B**) Expression trend of mature microRNAs in BN and CM. Before-after blot shown the same expression trend between mature microRNAs. (**C**). Mature microRNA abundance distribution in BN and CM. A stacked bar chart illustrates a different mature microRNA abundance distribution between BN and CM. Mature microRNAs are sorting based on their expression in BN.

The second class is formed by miRNAs with the same trend of variation for all their isomiRs in CM vs. BN, but with different abundance distribution between CM and BN. miR-101-3p and miR-27b-3p belong to this class. Almost all variants and canonical miR-101-3p have a significantly lower expression in CM (Figure 4A,B). Mature microRNAs of miR-101-3p are not distributed in the same way in CM and BN, though. As represented in Figure 4C, we can observe that the major expressed isomiR is miR-101-3p|0|0(+1U) in CM and miR-101-3p|−1|−1 in BN. All 9 isomiRs and canonical form of miR-27b-3p are downregulated in CM compared to BN with a significant difference in most of the cases (Figure 4A,B). Focusing on abundance distribution of the different variants and canonical miRNA, we observed that miR-27b-3p|0|−1 is the most expressed form in CM, while the canonical miRNA is the most expressed mature miRNA in BN (Figure 4B,C).

#### 2.5.3. IsomiRs with Opposite Trend in CM vs. BN and Similar Relative Abundance

**Figure 5:**
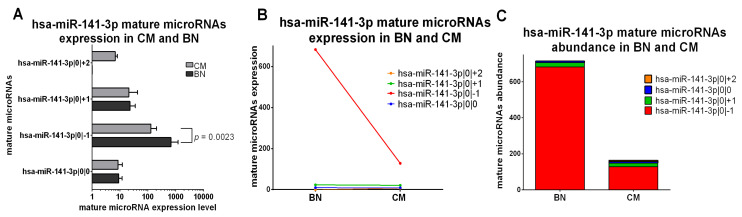
Group 3: IsomiRs with opposite trends in 20 CM vs. 3 BN and similar relative abundance. (**A**) Mature microRNA expression in BN and CM. All mature microRNAs are represented in a separated bar chart (mean). Bar indicates standard deviation. Mann-Whitney t test was performed to compared expression level in BN and CM. *p* values are shown when the difference is statistically significant. (**B**) Expression trend of mature microRNAs in BN and CM. Before-after blot shown a different expression trend between mature microRNAs. (**C**) Mature microRNA abundance distribution in BN and CM. The stacked bar chart illustrates the same mature microRNA abundance distribution between BN and CM. Mature microRNAs are sorting based on their expression in BN.

The only miRNA of the third class is miR-141-3p. This isomiR is downregulated in CM compared to BN (miR-141-3p|0|−1, *p* value = 0.0023), but the canonical miRNA and miR-141-3p|0|+1 have a comparable expression in CM and BN. miR-141-3p|0|+2 is only detectable in CM samples (Figure 5A,B). The abundance distribution is similar between CM and BN, in fact miR-141-3p|0|−1 isomiR is the most expressed mature microRNA in both groups (Figure 5C).

#### 2.5.4. IsomiRs with Opposite Trend in CM vs. BN and Different Relative Abundance

**Figure 6:**
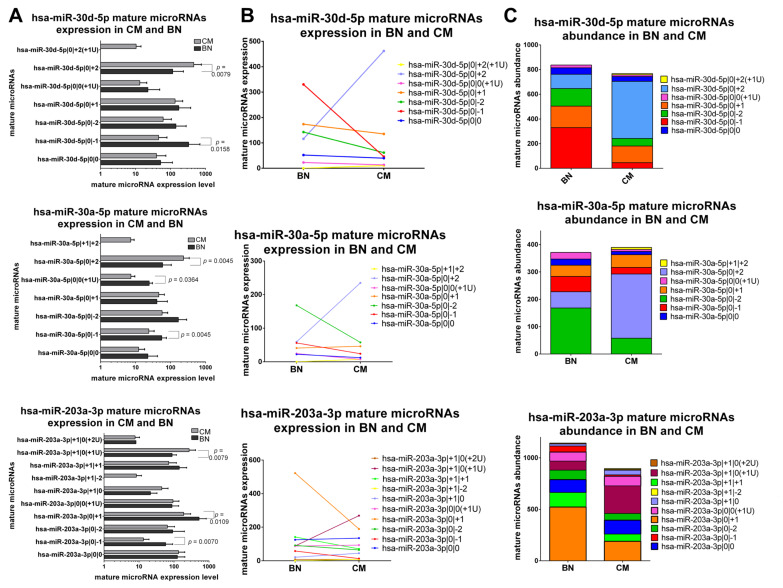
Group 4: IsomiRs with the opposite trend in 20 CM vs. 3 BN and different relative abundance. (**A**) Mature microRNA expression in BN and CM. All mature microRNAs are represented in a separated bar chart (mean). Bar indicates standard deviation. A Mann-Whitney t test was performed to compared expression level in BN and CM. *p* values are shown when the difference is statistically significant. (**B**) Expression trend of mature microRNAs in BN and CM. Before-after blot shown a different expression trend between mature microRNAs. (**C**) Mature microRNA abundance distribution in BN and CM. The stacked bar chart illustrates a different mature microRNA abundance distribution between BN and CM. Mature microRNAs are sorting based on their expression in BN.

The last class comprises three miRNAs, namely miR-30a-5p, miR-30d-5p and miR-203a-3p, which show different isomiRs abundance distribution in CM and BN and a different expression trend between isomiRs and the canonical form (Figure 6A–C).

In the case of miR-30a-5p, we identified six different isomiRs that are expressed in CM and BN. The most expressed variant in CM is miR-30a-5p|0|+2, while in BN is miR-30a-5p|0|−2 (Figure 6C). Looking at variants that are differently expressed between CM and BN, we noticed that one is upregulated (miR-30a-5p|0|+2), while two are downregulated (miR-30a-5p|0|−1, miR-30a-5p|0|0(+1U)) in CM (Figure 6A,B).

Also miR-30d-5p have six detected isomiRs. One of the isomiRs is detected only in CM (miR-30d-5p|0|+2(+1U)), and the canonical form and several isomiRs do not show differences between CM and BN. However, there are two isomiRs that have a significant and opposite trend: miR-30d-5p|0|−1 is under expressed in CM, while miR-30d-5p|0|+2 is overexpressed in CM (Figure 6A,B). Moreover, miR-30d-5p|0|−1 and miR-30d-5p|0|+2 are also the most expressed isomiR in CM and BN, respectively (Figure 6C).

Looking at the expression trend in CM and BN of the nine isomiRs of miR-203a-3p, only three of them have different expression. MiR-203a-3p|0|−1 and miR-203a-3p|0|+1 are downregulated in CM, while miR-203a-3p|+1|0(+1U) is upregulated in CM (Figure 6A,B). Furthermore, isomiR abundance distribution is different. In fact, the most expressed variant in BN is miR-203a-3p|0|+1, while in CM is miR-203a-3p|+1|0(+1U) (Figure 6C).

### 2.6. IsomiR Classification in Fresh-Frozen Primary Melanoma and Metastasis from TCGA Database

In order to identify isomiRs potentially associated with tumor progression, we interrogated the SKCM cohort of The Cancer Genome Atlas (TCGA) database. The TCGA database collects clinical and molecular data from fresh-frozen tissues, including smallRNA seq data. We explored the contribution of isomiRs in 323 melanoma patients, including 63 primary cutaneous melanomas (CM), and 260 melanoma metastases (MM), and assessed the association of isomiR expression with melanoma mutated genes. We detected a total of 3690 mature microRNAs, including 474 canonical microRNAs and 3216 isomiRs (Supplementary Table S4). Among the canonical miRNAs, only 85 miRNAs did not have any detected isomiR, while 389 canonical microRNAs had at least one co-expressed isomiR. Again, we observed the presence of orphan isomiRs (*n* = 258). However, for the majority of isomiRs, the canonical forms are detectable (*n* = 2958). The classification in isomiR types is reported in Table 4. The most frequent isomiR group is the end-site type. Interestingly, in this isomiRs list we showed the presence of a 5’ non-templated addition group, namely isomiRs with the addition of one or more nucleotides in the 5’ end, which did not match with the stem-loop sequence.

In addition, we found four novel miRNAs and three isomiRs of novel miRNAs. These novel miRNAs have been previously detected by examining small RNA-seq samples [28] (Supplementary Table S4).

### 2.7. Identification of the isomiRs More Expressed Than the Canonical Form in Fresh Primary Melanoma Samples from TCGA

To identify the most abundant isomiRs in fresh-frozen melanomas we investigated isomiRs that showed a higher or an equal expression level than the corresponding canonical mRNA form in primary melanomas from the TCGA SKCM cohort, which includes mostly stage II and stage III fresh primary melanoma.

We considered the average expression of mature microRNAs in 63 CM and we calculated the expression ratio between 2958 isomiRs and the corresponding canonical miRNA. We obtained a wide set of ratios that are reported in Supplementary Table S5, and 1373 of them showed a ratio higher than 1. Among these 1373 isomiRs, there were also 21 of the isomiRs identified in early-stage FFPE melanomas (mostly stage I melanomas) reported in Table 2, confirming the identification of these isomiRs also in fresh melanoma tissue and late-stage melanomas (Table 5).

### 2.8. IsomiR Expression Contributes to Distinguish Primary Melanoma and Metastasis

We compared the mature microRNA profile of 63 fresh-frozen CM and 260 MM samples from the TCGA database using an unpaired t-test (Benjamini-Hochberg correction, fold change > 1.5, adjusted *p* value < 0.05). We obtained a list of 332 mature microRNAs differentially expressed between the two groups. Specifically, 121 are downregulated and 211 are upregulated in MM vs. CM (Supplementary Figure S1, Supplementary Table S6). As for the type of mature miRNAs, 36 are canonical miRNAs, while the remaining 296 are isomiR variants. The most representative isomiR type is end-site group with 175 miRNA variants. Other isomiRs belong to start-site (27), 3’ non-templated addition (17), shifted (7). The remaining 70 are mixed isomiRs, specifically, 37 isomiRs that differentiate from the canonical form in the start-site and end-site; 29 in the end-site and 3’ non-template addition, 3 start-site and 3’ non-template addition, and 1 present different start-site, end-site and 3’ non-template addition.

Some isomiR variants show an opposite trend in CMs and MMs than their canonical miRNA. Specifically, miR-101-3p, miR-146b-3p, miR-148a-3p, miR-15a-5p, miR-16-5p, miR-181a-2-3p, miR-181a-5p, miR-181b-5p, miR-21-5p, miR-22-3p, miR-23a-5p, miR-23b-3p, miR-24-3p, miR-30e-5p, miR-361-3p, and miR-500a-3p have at least two variants with opposite trends (Supplementary Table S5, Supplementary Figure S2).

MMs have an upregulated isomiR (miR-146b-3p|0|−1) and a downregulated isomiR (miR-146b-3p|−1|−2) of miR-146b-3p. Similarly, also for miR-15a-5p, miR-16-5p and miR-181a-5p there is an upregulated isomiR (miR-15a-5p|0|−2, miR-16-5p|0|−2, miR-181a-5p|0|−3, respectively), and a downregulated isomiR (miR-15a-5p|0|+2, miR-16-5p|0|+3, miR-181a-5p|0|+2, respectively).

miR-148a-3p presents several differentially expressed isomiR variants, of which five are upregulated: (miR-148a-3p|+1|0, miR-148a-3p|+1|−1, miR-148a-3p|0|−1, miR-148a-3p|0|−2, and miR-148a-3p|0|−3), and two are downregulated (miR-148a-3p|0|+2, miR-148a-3p|0|0(+2U)) in MM compared to CM. Two upregulated isomiRs of miR-361-3p (miR-361-3p|0|−2, miR-361-3p|0|−3) and two downregulated isomiRs (miR-361-3p|+1|+2, miR-361-3p|0|+2) have been found in MM compared to CM.

Most of the mature microRNAs of miR-181a-2-3p and miR-181b-5p are downregulated in MM (miR-181a-2-3p|0|+1, miR-181a-2-3p|−1|0, miR-181a-2-3p|−1|−1; miR-181b-5p|0|+1, miR-181b-5p|0|+2(+1U), miR-181b-5p|0|0), while only one is upregulated (miR-181a-2-3p|0|−3, miR-181b-5p|0|−3). Similarly, miR-23b-3p and miR-500a-3p have only one upregulated mature microRNA (miR-23b-3p|0|−4, miR-500a-3p|−1|−3), while the others are downregulated in MM (miR-23b-3p|+1|0, miR-23b-3p|+1|0(+1U), miR-23b-3p|0|0, miR-23b-3p|0|0(+1U), miR-23b-3p|0|−1(+1U), miR-23b-3p|0|−1(+2U); miR-500a-3p|0|+1(+1U), miR-500a-3p|−1|+1, miR-500a-3p|−1|0, miR-500a-3p|−1|−4).

On the other hand, for miR-21-5p, miR-22-3p, miR-24-3p and miR-30e-5p there are mostly upregulated isomiRs (miR-21-5p|+2|0, miR-21-5p|+4|0, miR-21-5p|0|−2, miR-21-5p|0|−3, miR-21-5p|0|−4; miR-22-3p|+2|0, miR-22-3p|0|−2, miR-22-3p|0|−3, miR-22-3p|0|−4; miR-24-3p|0|−3, miR-24-3p|0|−3(+1U); miR-24-3p|0|−4, miR-30e-5p|+1|−2, miR-30e-5p|0|−2, miR-30e-5p|0|−3) and only one downregulated (miR-21-5p|0|+2, miR-22-3p|0|+1(+1U), miR-24-3p|0|0(+2U), miR-30e-5p|+1|+4, respectively) in MM compared with CM. Three isomiR variants of miR-101-3p are differently expressed. Specifically, miR-101-3p|0|−2 and miR-101-3p|−1|−2 are upregulated, while miR-101-3p|0|0(+2U) is downregulated in MM compared to CM. In addition, miR-23a-3p presents three upregulated isomiRs (miR-23a-3p|+1|−1, miR-23a-3p|0|−2, miR-23a-3p|0|−3) and two downregulated isomiRs (miR-23a-3p|0|+1(+2U), miR-23a-3p|0|+2(+1U)).

### 2.9. Identification of isomiRs Associated with Driver GeneMutations in TCGA Samples

We obtained the mutation status of BRAF, NRAS and NF1 for the melanoma samples from the TCGA database. We reported the mutation status of the three genes (when this information was available), in Supplementary Table S7. The most frequent mutation in BRAF is the missense mutation V600E, which is present in 145 samples. Among these 145 samples, 21 samples have also the mutation V600M. NRAS mutations are mostly missense mutation in Q61- position. Specifically, we found 33 samples with Q61R, 28 samples with Q61K, 11 samples with Q61L and five samples with Q61H.

NF1 presents a wide variety of different mutations and different types of mutations, including missense, stop codon, frameshift, and splice mutations. We analyzed the isomiR differential expression in mutated NF1, BRAF and NRAS compared to wild type samples.

#### 2.9.1. Identification of Mature microRNAs Associated with Mutated NF1

The NF1 mutation status was available for 66 melanoma samples: 51 patients presented at least one mutation in NF1, and 15 patients had wild type NF1. Comparing the mature microRNA expression in melanomas with NF1mut vs. NF1wt (T Test unpaired unequal variance (Welch), adjusted *p* value < 0.1), we found a list of five differentially expressed mature microRNAs (Table 6).

#### 2.9.2. Identification of Mature microRNAs Associated with Mutated BRAF

Among 187 samples that were tested for BRAF mutations, 19 samples had no BRAF mutations and 168 samples had at least one mutation in BRAF. The most frequent mutation of BRAF is in position V600- (149 patients): V600E (118), V600E + V600M (21), V600M + V600G (3), V600E + V660V (2), V600E + other mutations (5). We investigated the association between isomiR expression and BRAF mutation, comparing mutated vs. wild type BRAF. We found a list of 44 mature microRNAs differentially expressed. Specifically, two isomiRs are downregulated (hsa-miR-181a-2-3p|0|+1, hsa-miR-92a-3p|0|+1(+1U)), while the remaining 42 are upregulated in BRAF mutated melanoma patients (Table 6).

We identified the mature microRNAs that are specifically altered in BRAF (V600E) melanoma (Table 7). Interestingly, melanomas with exclusively V600E mutation and melanomas with both V600E and V600M BRAF mutations present 7 differentially expressed mature microRNAs. Specifically, V600E|V600M BRAF mutated melanomas downregulated let-7b-3p|0|−1(+1U), miR-100-5p|0|0(+1U), miR-125b-5p|0|0, miR-221-3p|0|−1, and upregulated let-7a-5p|+1|−2, miR-1247-5p|0|−1, miR-219a-1-3p|0|0 compared to V600E BRAF mutated melanomas.

#### 2.9.3. Identification of Mature microRNAs Associated with Mutated NRAS

In the TCGA database, 111 melanoma samples were tested for NRAS mutations. 26 melanomas have wild type NRAS, while 85 have at least one mutation in NRAS. Comparing the mature microRNA expression in mutated and wild type NRAS, we identified six differentially expressed mature microRNAs (Table 8).

## 3. Discussion

The miRBase database provides a single mature sequence for each miRNA, which is usually the sequence with the highest coverage reported in small RNA sequencing experiments. However, we discovered that in both FFPE and fresh-frozen melanoma samples several isomiRs are more abundant than the canonical forms, being up to 50 times more expressed in FFPE early-stage melanoma samples. We observed that end-site isomiRs are the most represented group in FFPE and fresh melanomas. Similarly, in colon cancer it was observed that 3’ isomiRs are the most frequent microRNA variants [29].

In the literature, there is a concern that sequencing and/or mapping artefacts may result in an overrepresentation of isomiRs [30]. However, previous studies have shown that isomiRs represent actual molecules rather than sequencing artifacts. First, by using aggressive filtering, most categories of isomiRs are detected at levels above thresholds comparable to their canonical miRNA sequence. In many cases, the isoform is present at increased levels as compared to the canonical sequences [19]. Furthermore, various studies have shown that isomiR profiles across tissues differ. In other words, the most abundant isoform does differ across tissues and disease states. This suggests that isomiR biogenesis may be a regulated process [31]. Furthermore, sequence library preparation could cause miRNA degradation at the miRNA ends, resulting in the identification of isomiRs. If true, these effects cannot explain the bias of the heterogeneity between the two miRNA ends in which the 3’-ends are usually more diverse than the 5’-ends [32]. Additionally, a significant number of isomiRs are also longer and more abundant than the canonical miRNA. These observations suggest that isomiRs are not experimental artifacts but rather true biological events. Our sequence analysis pipeline uses isoMiRmap [27]. In a deterministic and exhaustive manner, isoMiRmap comprehensively reports all isomiRs whose sequences exist within known miRNA sequences. Additionally, this approach identifies 3’-non-templated isomiRs.

Similarly to canonical miRNAs, isomiR expression can discriminate human cancers, [24]. In addition, recent studies have demonstrated that isomiRs could be used as prognostic and diagnostic biomarkers in various cancers and related subtypes; in fact, they can differentiate between healthy and non-healthy individuals [33]. To analyze the contribution of isomiRs to melanoma pathogenesis, we compared the isomiR profile of benign nevi and early-stage primary cutaneous melanoma FFPE samples, and of primary cutaneous melanoma and melanoma metastasis of fresh-frozen samples.

A differential expression of isomiRs was observed between benign nevi and early-stage melanoma FFPE samples; specifically, 55 mature microRNAs (including the canonical form and all isoforms) were differently expressed. Interestingly, for some miRNAs only the isomiR(s) was differently expressed and not the canonical miRNA. Among these 55 mature microRNAs, we identified 16 isomiRs that were more abundant than their canonical counterpart. We focused on these 16 isomiRs, which were classified in four groups according to the expression trend in tumors and miRNA/isomiR relative abundance distribution.

Among the mature microRNAs with similar expression trends and similar abundance distribution in nevi and early-stage melanomas, we found miR-19b-3p, miR-27a-3p, miR-29a-3p and miR-222-3p. Despite our data that showed that miR-19b-3p and its isomiR are downregulated in early-stage FFPE melanoma, an in vitro study showed that miR-19b-3p expression is higher in most melanoma cell lines compared with normal melanocytes, where it promotes cell proliferation [34]. The literature suggests that melanoma tissues upregulate miR-27a-3p and its expression is positively correlated with tumor stage and lymph node metastasis of melanoma tissues. The downregulation of miR-27a-3p induces autophagy and apoptosis of melanoma cells [35]. No data exist about the differential expression of this miRNA between nevi and melanoma. We found that miR-29a-3p expression is lower in early-stage melanoma. This data is in agreement with the literature; in fact it was observed that the overexpression of miR-29a-3p inhibits proliferation, migration, and invasion, and promotes apoptosis by blocking Wnt/β-catenin and NF-κB pathways [36]. The higher expression of miR-222-3p and its isomiRs in early-stage melanoma suggests an oncogenic role, in fact miR-222-3p belongs to the miR-221 family, which is an important oncogenic microRNA family involved in the progression of melanoma and have a key role in EMT [37]. For this class of isomiRs, we hypothesized a cooperative effect of the isomiRs on the target genes.

In the second class we identified miR-101-3p and miR-27b-3p mature microRNAs, which have the same expression trend but different abundance distribution in benign nevi and early-stage melanomas. All their mature microRNAs are downregulated in melanoma. These results agree with previous studies describing these two canonical miRNAs as tumor suppressor miRNAs. miR-101-3p directly targets MITF and EZH2, resulting in inhibition of invasion and proliferation. In addition, low expression of miR-101-3p correlated with a poor survival in stage IV melanoma patients [38]. Canonical miR-27b-3p inhibits the development of melanoma by targeting MYC [39].

We identified a specific isomiR of miR-141-3p, miR-141-3p|0|−1, which is downregulated in early-stage melanoma, while the canonical miRNA is not differentially expressed. In both groups, namely benign nevi and early-stage melanoma, this specific isomiR is the most abundant variant. This seems to suggest that only this isomiR could have a protective role, and not the canonical form or the other variants. The canonical miRNA was found to inhibit the proliferation of melanoma cells [40]. 

Our analysis revealed that miR-30a-5p, miR-203a-3p and miR-30d-5p showed a different expression trend for at least two isoforms, and a different mature microRNA prevalence in benign nevi and early-stage melanomas. The canonical miRNAs of these isomiRs have been extensively studied in melanoma. MiR-30a-5p is considered a tumor suppressor microRNA in melanoma. In fact, miR-30a-5p is normally downregulated in melanoma tissues and cell lines. The overexpression of miR-30a-5p leads to a significant inhibition of proliferation, migration, and invasion of melanoma cells. Moreover, miR-30a-5p in vivo delays tumor growth [41] and inhibits metastasis [42]. In our analysis, the canonical miR-30a-5p is more expressed in benign nevi than early-stage melanomas, although the difference is not statistically significant. We can speculate that the tumor suppressor effects derive from the cumulative activity of all the isoforms.

MiR-203a-5p is an important tumor suppressor miRNA in melanoma, involved in the regulation of proliferation, cell cycle, migration, and invasion [43,44]. The expression of canonical miR-203a-3p is downregulated in melanoma, especially in metastatic melanoma. Low miR-203a-3p expression is associated with poor overall survival [45]. MiR-30d-5p acts as an oncomiR in melanoma. High expression of miR-30d-5p in melanoma is associated with stage, metastatic potential, shorter time to recurrence, and reduced overall survival. miR-30d-5p induces metastatic behavior of melanoma cells [46]. In this analysis, BN and melanomas show similar expression levels of miR-203a-3p and miR-30d-5p canonical miRNAs, which are probably linked with the early stages of the melanomas, but several isoforms of miR-30 are significantly downregulated in tumors.

To study the isomiR contribution to melanoma progression, we took advantage of data available from TCGA. The TCGA database provides clinical and molecular information from fresh tumor tissues, including smallRNA sequencing data from primary melanoma and melanoma metastasis (Skin cutaneous melanoma, SKCM). In the SKCM cohort, the primary tumors are mostly of advanced stages (stage II and stage III). Primary melanoma and melanoma metastasis present strong differences in the isomiR profile; in fact, 121 mature microRNAs are downregulated and 211 are upregulated in melanoma metastases compared with primary melanomas. Among them, miR-101-3p, miR-146b-3p, miR-148a-3p, miR-15a-5p, miR-16-5p, miR-181a-2-3p, miR-181a-5p, miR-181b-5p, miR-21-5p, miR-22-3p, miR-23a-5p, miR-23b-3p, miR-24-3p, miR-30e-5p, miR-361-3p, and miR-500a-3p have at least two variants with opposite trends in primary melanoma and metastasis.

Mutations in key genes of the MAPK/ERK pathway, namely NF1, NRAS and BRAF, identify three main molecular subtypes of melanoma. Several microRNAs are known to affect or to be affected by the altered activation of this pathway in melanoma, and some of them are responsible for drug resistance [47]. Since the study of the expression of isomiRs in relation to the mutation status of these proteins is missing in the literature, we focused on the different isomiR profiles associated with the different mutation status of NF1, BRAF and NRAS.

We collected information on the mutation status of BRAF, NRAS and NF1 genes for SKCM TCGA samples and investigated the association between isomiR expression and the most clinically relevant mutations in melanoma.

Somatic mutations of BRAF gene have been found in almost 47% of sporadic cutaneous melanomas. The most frequent mutation is the V600E (56%), while other activating mutation at codons 600 and 601, such as V600K V600R V600D K601E, were less frequent (20%, 2%, 1%, 2% respectively). NRAS mutations were found in 28% of the tumors and almost all of them occurred at “hotspot” codons 12, 13 or 61 (6%, 4%, 86% respectively) [48]. We identified isomiRs differentially expressed in the NF1, BRAF and NRAS mutation groups, suggesting that the identified isomiRs could have a suppressive, promotive or cooperative role in the MAPK pathway.

Several canonical miRNAs of isomiRs that are differently expressed in mutated BRAF samples were already studied in relation to BRAF. It was observed that Vemurafenib, a BRAF inhibitor, alters the miRNA expression in melanoma cells, leading to an upregulation of miR-181a-2-3p [49]. MiR-181c-5p was found to be associated with the BRAF mutation in thyroid follicular adenomas. Indeed, its expression is upregulated in RAS- or BRAF-mutated follicular adenomas compared to wild-type tumors [50]. However, its role in melanoma has yet to be established.

Among the miRNAs whose isoforms are associated with BRAF mutations, let-7a-5p and let-7b-5p are well known important tumor suppressor miRNAs which are downregulated in melanoma and inhibit proliferation and migration of melanoma cells [51]. miR-143-3p acts as an inhibitor of migration and proliferation and an inducer of apoptosis in melanoma cancer cells in vitro [52]. miR-154-5p represses cell proliferation and metastasis in melanoma [53], while miR-330-5p, miR-342-5p, and miR-942-5p inhibit cell proliferation and invasion in cutaneous malignant melanoma [54,55,56].

It has been described that B-Raf/MKK/ERK can upregulate miRNAs in melanoma cells, specifically the miR-17-92 microRNA family [57]. We observed a remarkable upregulation of several isomiRs from the miR-17-92 cluster in NRAS mutant melanomas.

IsomiR expression validation with methods other than NGS is still an open issue, given the lack of commercial kits or largely recognized approaches. The most promising technique to quantify miRNA variants is Dumbbell PCR. Honda et al. developed this method to quantify specific small RNA variants with a single nucleotide resolution at terminal sequences (5’ and 3’) [58]. In our previous work, we combined two different commercial kits to quantify the sum of 5’ and 3’ isoforms and the canonical miRNA [26]. The development of reliable assays will be of the utmost importance for further functional studies involving isomiRs.

## 4. Materials and Methods

### 4.1. FFPE Samples and Small-RNA Sequencing Data

Patients characteristics and experimental procedure are described in our previous work [26]. In this paper, we used small-RNA sequencing data from three benign nevi (BN) and 20 melanomas (CM).

Samples were obtained from the melanoma center of the Dermatology Unit at Bologna University Hospital. The study was approved by Comitato Etico Indipendente di Area Vasta Emilia Centro—CE-AVEC, Emilia-Romagna Region (number 417/2018/Sper/AOUBo). Histopathologic specimens were evaluated by two dermato-pathologists and classified into benign nevi and melanomas. BN includes patients with no prior diagnosis of CM or non-melanoma skin cancer and a follow-up of at least 10 years. CM includes 19 patients with superficial cutaneous melanoma (stage I, Breslow range = 0.3–.), and one patient with nodular cutaneous melanoma (stage II, Breslow = 2.5). Patient specimens were formalin-fixed and paraffin-embedded (FFPE), which were used to RNA extraction using the miRNeasy FFPE kit (Qiagen, Hilden, Germany) according to the manufacturer’s instructions. The 23 small-RNA libraries were generated using TruSeq Small RNA Library PrepKit v2 (Illumina, San Diego, CA, USA, RS-200-0012/24/36/48), according to the manufacturer’s directions. Raw base-call data were demultiplexed using an Illumina BaseSpace Sequence Hub and converted to FASTQ format [26]. The small RNA sequencing raw data (FASTQ format) are available at the European Nucleotide Archive (ENA) with the following accession number: PRJEB35819.

### 4.2. IsomiR Analysis of TCGA SKCM Cohort

We collected isomiR data and mutation status of BRAF, NRAS and NF1 from 323 melanoma fresh-frozen samples from The Cancer Genome Atlas (TCGA), including 63 primary cutaneous melanomas (CM) and 260 melanoma metastases. In the TCGA database, ”metastatic melanoma” refers to the metastatic tissue and not to the primary melanoma that have metastasized. To avoid misunderstanding, we preferred to call this group melanoma metastasis (MM), instead of metastatic melanoma. CMs are mostly II or III stage (37 samples are stage II, 19 are stage III, 3 are stage IV, and for four of them the stage was not reported); and the biopsy site was: 58% lymph nodes, 21% connective, subcutaneous and other soft tissues, 17% Skin and 4% other.

The NF1 mutation gene status was available for 66 melanomas (51 samples have at least one mutation, and 15 are the wild type). BRAF mutation gene status was available for 187 melanomas (168 have at least one mutation, 19 are wild type). NRAS mutation gene status was available for 111 melanomas (85 have at least one mutation, while 26 are wild type).

All detailed data of this melanoma dataset are available on National Cancer Institute GDC Data Portal site (https://portal.gdc.cancer.gov, accessed on 1 June 2021).

### 4.3. IsomiR Identification

The mapping of sequence reads and isomiR quantification was performed using the isoMiRmap tools [27]. isoMiRmap identifies and quantifies all isomiRs through the direct processing of short RNA-sequencing datasets. Briefly, for each short RNA-sequence dataset, the sequence reads were quality trimmed using the Cutadapt tool [59], keeping only those reads between 18 and 26 nts in length. Each resulting read is subsequently compared to a “lookup” table containing all possible isomiRs from miRbase release 22 hairpin sequences. All sequences are required to have exact matches to the isomiR lookup table, after which they are then counted and quantified in read per million (RPM). This approach identifies 5’-isomiRs, 3’-isomiRs, 5’-and-3’ mixed isomiRs, as well as 3’ non-templated post-transcriptional additions. This approach was used for both those datasets generated here and those samples represented in TCGA.

### 4.4. IsomiR Labeling and Classification

The annotation system developed by Loher et al. was used to label isomiRs [32]. Briefly, nomenclature indicates the name of the canonical miRNA, 5’ end (start site), 3’ end (end-site) and the eventual insertion of uracil of the isomiR compared to the canonical miRNA sequence in miRBase database. Specifically, to define the start site and end site, zero indicates the same terminus of the canonical miRNA, while positive sign (+) or negative sign (-) followed by the number indicates the isomiR nucleotide shift in the 3’ or 5’ direction, respectively, when compared with miRBase microRNA sequence. IsomiR were classified in six different groups depending on the change from the canonical miRNA: start site isomiRs (those isomiRs that varies in the start site with deletion or addition of nucleotides that match with stem loop), end site isomiRs (those isomiRs that varies in the end site, with deletion or addition of nucleotides that match with stem loop), 5’ non-templated addition isomiRs (isomiRs with addition of nucleotides at the 5’ end, which do not match with stem loop), 3’ non-templated addition isomiRs (isomiRs with addition of nucleotides at the 3’ that do not match with stem loop sequence), shifted isomiRs (start site and end site are both shifted to 5’ or to 3’ direction, but the length of the isomiR is identical to the canonical miRNA), and mixed isomiRs (isomiRs with more than one variation).

### 4.5. Statistical Analysis

Normalized data were analyzed with GeneSpring GX software (Agilent Technologies). Differentially expressed mature miRNAs (canonical miRNAs + isomiRs) were identified using a fold change > 2 filter and moderated t-test (FDR 5% with Benjamini-Hochberg correction) in benign nevi vs. melanoma comparison (small-RNA seq data). For TCGA SKCM data, we applied a fold-change > 1.5 and t-test unpaired (FDR 5% with Benjamini-Hochberg correction) in primary melanoma vs. melanoma metastasis comparison. To analyze the association between isomiR and the melanoma-linked mutation, we performed an unpaired t-test with unequal variance (Welch correction) (FDR 5% with Benjamini-Hochberg correction). Graphpad Prism 6 (GraphPad Software, San Diego, CA, USA) was used to perform specific isomiR comparisons using the Mann-Whitney non-parametric test.

## 5. Conclusions

Tumor characterization at the small RNA level, when focusing only on canonical miRNAs, has the risk to overlook and lose important information that could improve our understanding of cancer pathogenesis. Here, we provided a comprehensive characterization of isomiR dysregulation in benign nevi, melanoma at different stages and sources, and melanoma metastasis. We showed that isomiRs can be more expressed than their canonical miRNA and also that different isoforms are modulated in different and sometimes opposite ways in normal and tumor samples, a fact that was not expected. We reported that specific isomiRs are associated with tumor mutation status, sometimes diverging from the trend of the canonical miRNA. Our results and observations about a non-random dysregulation of specific isomiRs in melanoma serve as the basis for further functional studies.

## Figures and Tables

**Table 1:** Mature microRNA classification in 23 formalin-fixed paraffin-embedded (FFPE) samples.

Mature miRNAs from Small RNA Seq of FFPE Samples			
Type	Sub-type	Total	
“canonical” miRNA	miRNA with isomiR(s)	90 (18.2%)	130 (26.3%)
	miRNA without isomiR	40 (8.1%)	
isomiR	isomiR with canonical miRNA	324 (65.6%)	364 (73.7%)
	orphan isomiR	40 (8.1%)	
Total			494 (100%)
Classification of canonical miRNAs with isomiR(s)			
Groups	Sub-groups	Total	
miRNA with 1 isomiR		25 (27.8%)	25 (27.8%)
miRNA with 2-3 isomiRs	2 isomiRs	14 (15.6%)	27 (30.0%)
	3 isomiRs	13 (14.4%)	
miRNA with 4-5 isomiRs	4 isomiRs	10 (11.1%)	21 (23.3%)
	5 isomiRs	11 (12.2%)	
miRNA with 6-13 isomiRs	6 isomiRs	6 (6.7%)	17 (18.9%)
	7 isomiRs	2 (2.2%)	
	8 isomiRs	2 (2.2%)	
	9 isomiRs	3 (3.4%)	
	10 isomiRs	2 (2.2%)	
	11 isomiRs	1 (1.1%)	
	13 isomiRs	1 (1.1%)	
Total			90 (100%)
Classification of isomiRs with canonical miRNA			
Groups	Sub-groups	Total	
End-site isomiR		199 (61.4%)	199 (61.4%)
Start-site isomiR		19 (5.9%)	19 (5.9%)
Shifted isomiR		9 (2.8%)	9 (2.8%)
3′ non-templated addition isomiR		43 (13.2%)	43 (13.2%)
Mixed isomiR	End-site + start-site	13 (4.0%)	54 (16.7%)
	End-site + 3′ non-templated addition	34 (10.5%)	
	Start-site + 3′ non-templated addition	6 (1.9%)	
	Shifted + 3′ non-templated addition	1 (0.3%)	
Total			324 (100%)

**Table 2:** List of isomiRs in 20 FFPE melanoma samples with an isomiR/canonical miRNA ratio greater than 1.

Mature microRNA Name	Normalized Expression isomiR (Average, *n* = 20)	Normalized Expression Canonical miRNA (Average, *n* = 20)	Ratio isomiR/Canonical miRNA
hsa-miR-222-3p|0|+3	170.56	3.17	53.84
hsa-miR-141-3p|0|−1	127.96	4.26	30.01
hsa-miR-30a-5p|0|+2	234.85	10.41	22.55
hsa-miR-222-3p|0|+4	48.31	3.17	15.25
hsa-miR-222-3p|0|+2	38.26	3.17	12.08
hsa-miR-30d-5p|0|+2	462.00	39.70	11.64
hsa-miR-200b-3p|0|+1	44.59	4.29	10.39
hsa-miR-125a-5p|0|−2	623.96	60.29	10.35
hsa-miR-30c-5p|0|+1	153.33	15.63	9.81
hsa-miR-200b-3p|0|+1(+1U)	33.28	4.29	7.76
hsa-miR-10b-5p|0|−1	2368.09	365.48	6.48
hsa-miR-30a-5p|0|−2	57.72	10.41	5.54
hsa-miR-19b-3p|0|−1	25.74	4.85	5.31
hsa-miR-199a-3p|0|−1	258.78	52.96	4.89
hsa-miR-26b-5p|0|+1	165.47	35.93	4.61
hsa-miR-30a-5p|0|+1	46.17	10.41	4.43
hsa-miR-27a-3p|0|−1	100.82	24.74	4.08
hsa-miR-30d-5p|0|+1	135.04	39.70	3.40
hsa-miR-221-3p|0|−1	39.05	11.80	3.31
hsa-miR-10a-5p|0|−1	594.23	197.37	3.01
hsa-miR-744-5p|0|−1	24.67	9.36	2.63
hsa-miR-30a-5p|0|−1	23.98	10.41	2.30
hsa-miR-27b-3p|0|−1	845.19	368.31	2.29
hsa-miR-101-3p|0|0(+1U)	47.54	21.42	2.22
hsa-miR-221-3p|0|−2	26.12	11.80	2.21
hsa-miR-509-3p|0|+1	88.74	40.27	2.20
hsa-miR-203a-3p|+1|0(+1U)	268.82	134.91	1.99
hsa-miR-101-3p|0|+1	39.31	21.42	1.83
hsa-miR-199a-3p|−1|−1	87.92	52.96	1.66
hsa-miR-29a-3p|0|−1	33.98	21.58	1.57
hsa-miR-30d-5p|0|−2	61.55	39.70	1.55
hsa-miR-203a-3p|0|+1	189.30	134.91	1.40
hsa-miR-142-5p|−2|−3	97.82	76.36	1.28
hsa-miR-125a-5p|0|−3	73.56	60.29	1.22
hsa-miR-205-5p|0|+1	1221.77	1044.60	1.17
hsa-miR-30d-5p|0|−1	45.81	39.70	1.15
hsa-miR-101-3p|−1|−1	23.09	21.42	1.08
hsa-miR-146b-5p|0|+1	35.42	33.14	1.07
hsa-miR-27a-3p|0|−2	25.16	24.74	1.02

**Table 3:** List of differentially expressed mature microRNAs in 20 FFPE early-stage melanoma (CM) and 3 FFPE benign nevus (BN) samples.

Mature miRNAs Differently Expressed in FFPE Samples: CM Vs BN			
Name	*p* Value (Corr)	Regulation	Log FC
Canonical miRNAs			
hsa-let-7b-5p|0|0	1.0E-02	up	2.73
hsa-miR-205-5p|0|0	9.3E-03	up	2.04
hsa-miR-143-3p|0|0	4.2E-02	up	1.50
hsa-miR-27a-3p|0|0	7.1E-03	down	−1.60
hsa-miR-192-5p|0|0	3.9E-02	down	−1.61
hsa-miR-99a-5p|0|0	2.1E-02	down	−1.77
hsa-miR-99b-5p|0|0	3.6E-02	down	−1.94
hsa-miR-28-3p|0|0	1.1E-02	down	−1.99
hsa-miR-181a-2-3p|0|0	5.8E-04	down	−2.05
hsa-miR-497-5p|0|0	3.4E-04	down	−2.27
hsa-miR-148a-3p|0|0	2.7E-03	down	−2.29
hsa-miR-30b-5p|0|0	3.3E-03	down	−2.47
hsa-miR-199a-3p|0|0	1.5E-05	down	−3.07
hsa-miR-101-3p|0|0	4.2E-05	down	−3.08
hsa-miR-199a-5p|0|0	8.9E-07	down	−3.21
hsa-miR-27b-3p|0|0	3.2E-06	down	−3.38
hsa-miR-125b-5p|0|0	9.6E-07	down	−3.98
hsa-miR-29a-3p|0|0	8.6E-09	down	−6.42
isomiRs			
hsa-let-7b-5p|0|+1	5.6E-03	up	2.54
hsa-miR-30d-5p|0|+2	7.2E-03	up	2.25
hsa-miR-30a-5p|0|+2	1.3E-03	up	2.17
hsa-miR-222-3p|0|+3	9.0E-03	up	2.00
hsa-miR-143-3p|0|0(+1U)	2.6E-02	up	1.71
hsa-miR-143-3p|0|−1	4.5E-02	up	1.70
hsa-miR-203a-3p|+1|0(+1U)	4.2E-02	up	1.46
hsa-miR-199a-3p|+1|−1	2.0E-02	down	−1.19
hsa-miR-30a-5p|0|−1	1.7E-02	down	−1.28
hsa-miR-30a-5p|0|−2	4.5E-02	down	−1.36
hsa-miR-26a-5p|+3|0	4.3E-02	down	−1.44
hsa-miR-103a-3p|0|−1(+1U)	3.2E-02	down	−1.57
hsa-miR-27b-3p|0|0(+1U)	5.0E-02	down	−1.57
hsa-miR-26a-5p|0|−1	2.0E-02	down	−1.64
hsa-miR-148a-3p|0|+1	3.2E-02	down	−1.73
hsa-miR-148a-3p|0|+2	1.7E-02	down	−1.75
hsa-miR-27b-3p|0|−1	2.7E-02	down	−1.78
hsa-miR-23b-3p|+3|−2	6.8E-03	down	−1.87
hsa-miR-148a-3p|0|0(+1U)	1.2E-02	down	−1.90
hsa-miR-26a-5p|0|0(+2U)	1.6E-04	down	−2.06
hsa-miR-27a-3p|0|−2	3.1E-03	down	−2.10
hsa-miR-101-3p|0|0(+1U)	2.7E-03	down	−2.12
hsa-miR-30e-5p|0|+1	4.8E-04	down	−2.35
hsa-miR-141-3p|0|−1	3.8E-03	down	−2.36
hsa-miR-30d-5p|0|−1	8.5E-03	down	−2.46
hsa-miR-99b-5p|0|−1	1.1E-02	down	−2.53
hsa-miR-199a-5p|0|−1	6.1E-06	down	−2.79
hsa-miR-27a-3p|0|−1	1.3E-03	down	−2.81
hsa-miR-19b-3p|0|−1	2.6E-04	down	−3.02
hsa-miR-101-3p|0|+1	3.4E-05	down	−3.15
hsa-miR-27b-3p|0|−2	3.4E-05	down	−3.23
hsa-miR-100-5p|0|−1	1.3E-03	down	−3.50
hsa-miR-27b-3p|0|+1	4.7E-06	down	−3.51
hsa-miR-27a-3p|0|−3	9.6E-07	down	−3.89
hsa-miR-27b-3p|0|−1(+1U)	1.2E-06	down	−3.95
hsa-miR-101-3p|−1|−1	1.2E-06	down	−4.70
hsa-miR-29a-3p|0|−1	4.0E-08	down	−5.79

**Table 4:** Mature microRNA classification in 323 Fresh-frozen Melanoma from TCGA SKCM cohort.

Mature miRNAs and Novel miRNAs from TCGA			
Type	Sub-type	Total	
”canonical” miRNAs	miRNA with isomiR	389 (10.52%)	474 (12.82%)
	miRNA without isomiR	85 (2.30%)	
isomiRs	isomiR with canonical miRNA	2958 (80.01%)	3216 (86.98%)
	orphan isomiR	258 (6.97%)	
novel miRNAs	with isomiR	1 (0.03%)	4 (0.11%)
	without isomiR	3 (0.08%)	
isomiRs of novel miRNAs	with novel miRNA	1 (0.03%)	3 (0.09%)
	without novel miRNA	2 (0.06%)	
Total			3697 (100%)
IsomiR classification			
Groups	Sub-groups	Total	
End-site isomiRs		1271 (39.52%)	1271 (39.52%)
Start-site isomiRs		341 (10.60%)	341 (10.60%)
Shifted isomiRs		187 (5.82%)	187 (5.82%)
3’ non-templated addition isomiRs		206 (6.41%)	206 (6.41%)
5’ non-templated addition isomiRs		3 (0.09%)	3 (0.09%)
Mixed isomiRs	start-site + end-site	510 (15.86%)	1208 (37.56%)
	start-site + 3’ non-templated addition	84 (2.61%)	
	end-site + 3’ non-templated addition	465 (14.46%)	
	start-site + end-site + 3’ non-templated addition	94 (2.92%)	
	5’ non-templated addition + end-site	9 (0.28%)	
	shifted + 3’ non-templated addition	46 (1.43%)	
***Total***			3216 (100%)

**Table 5:** List of isomiRs with an isomiR/canonical miRNA ratio greater than 1 in 63 fresh-frozen and 20 FFPE primary melanoma samples at different stages.

Mature microRNA Name	Ratio isomiR/Canonical miRNA in 63 Late-Stage CM Samples	Ratio isomiR/Canonical miRNA in 20 Early-Stage CM Sample
hsa-miR-101-3p|0|0(+1U)	1.21	2.22
hsa-miR-125a-5p|0|−2	2.22	10.35
hsa-miR-141-3p|0|−1	506.45	30.01
hsa-miR-142-5p|−2|−3	137.60	1.28
hsa-miR-146b-5p|0|+1	2.68	1.07
hsa-miR-199a-3p|0|−1	5.22	4.89
hsa-miR-199a-3p|−1|−1	1.14	1.66
hsa-miR-200b-3p|0|+1	2.41	10.39
hsa-miR-200b-3p|0|+1(+1U)	67,983.34	7.76
hsa-miR-203a-3p|+1|0(+1U)	16.12	1.99
hsa-miR-203a-3p|0|+1	6.59	1.40
hsa-miR-222-3p|0|+3	3.80	53.84
hsa-miR-222-3p|0|+4	57.90	15.25
hsa-miR-26b-5p|0|+1	15.51	4.61
hsa-miR-27a-3p|0|−1	2.53	4.08
hsa-miR-27a-3p|0|−2	1.68	1.02
hsa-miR-27b-3p|0|−1	522.43	2.29
hsa-miR-29a-3p|0|−1	9.61	1.57
hsa-miR-30c-5p|0|+1	18.70	9.81
hsa-miR-30d-5p|0|−1	1.37	1.15
hsa-miR-30d-5p|0|−2	33.78	1.55

**Table 6:** List of mature microRNAs associated with NF1 mutation in TCGA SKCM samples.

Comparison between NF1 Mutated and NF1 WT Samples					
Name	Sequence	Type	*p* (Corr) (Mutated NF1 Vs. Wild Type NF1)	Regulation (Mutated NF1 Vs. Wild Type NF1)	Log FC (Mutated NF1 Vs. Wild Type NF1)
hsa-miR-766-3p|0|−2	ACTCCAGCCCCACAGCCTCA	end-site	7.77E-02	up	1.17
hsa-miR-584-5p|−1|−2	ATTATGGTTTGCCTGGGACTG	mixed: start-site + end-site	8.38E-02	up	1.46
hsa-miR-378a-3p|−1|−1(+1U)	CACTGGACTTGGAGTCAGAAGGT	mixed: shifted + 3’non-templated addition	7.77E-02	down	−1.35
hsa-let-7d-5p|+1|−1	GAGGTAGTAGGTTGCATAGT	mixed: start-site + end-site	9.29E-02	up	0.70
hsa-miR-148a-3p|+3|−1	GTGCACTACAGAACTTTG	mixed: start-site + end-site	7.77E-02	up	1.03

**Table 7:** List of mature microRNAs associated with BRAF mutation in TCGA SKCM samples.

IsomiRs Differentially Expressed in Mutated vs. Wild Type (WT) BRAF Melanoma Samples					
Name	Sequence	Type	*p* (Corr) (mutated BRAF Vs. WT)	Regulation FC (mutated BRAF Vs. WT)	Log FC (mutated BRAF Vs. WT)
hsa-let-7d-3p|0|−2(+2U)	CTATACGACCTGCTGCCTTTTT	mixed: end-site + 3’ non-templated addition	4.40E-02	up	0.76
hsa-let-7i-3p|0|−2	CTGCGCAAGCTACTGCCTTG	end-site	2.12E-02	up	1.75
hsa-miR-100-5p|+1|−1	ACCCGTAGATCCGAACTTGT	mixed: start-site + end-site	2.14E-02	up	1.96
hsa-miR-100-5p|0|−1	AACCCGTAGATCCGAACTTGT	end-site	2.02E-02	up	2.00
hsa-miR-100-5p|0|−1(+2U)	AACCCGTAGATCCGAACTTGTTT	mixed: end-site + 3’ non-templated addition	7.63E-07	up	1.39
hsa-miR-106b-3p|+1|−1	CGCACTGTGGGTACTTGCTG	mixed: start-site + end-site	4.49E-03	up	0.81
hsa-miR-1247-5p|0|−1	ACCCGTCCCGTTCGTCCCCGG	end-site	2.02E-02	up	0.96
hsa-miR-125b-5p|0|+1	TCCCTGAGACCCTAACTTGTGAG	end-site	2.44E-04	up	0.86
hsa-miR-1307-3p|0|−2	ACTCGGCGTGGCGTCGGTCG	end-site	3.05E-02	up	0.80
hsa-miR-143-3p|0|−3(+1U)	TGAGATGAAGCACTGTAGT	mixed: end-site + 3’ non-templated addition	3.95E-02	up	1.06
hsa-miR-143-3p|−1|−1	CTGAGATGAAGCACTGTAGCT	shifted	4.64E-02	up	1.03
hsa-miR-143-3p|−1|−3	CTGAGATGAAGCACTGTAG	mixed: start-site + end-site	2.59E-02	up	1.21
hsa-miR-146a-5p|0|−4	TGAGAACTGAATTCCATG	end-site	3.11E-02	up	0.87
hsa-miR-154-5p|0|0	TAGGTTATCCGTGTTGCCTTCG	canonical	8.45E-03	up	0.94
hsa-miR-181a-2-3p|0|+1	ACCACTGACCGTTGACTGTACCT	end-site	2.17E-02	down	-1.57
hsa-miR-181c-5p|0|0	AACATTCAACCTGTCGGTGAGT	canonical	8.45E-03	up	1.07
hsa-miR-199a-3p|+3|−1	GTAGTCTGCACATTGGTT	mixed: start-site + end-site	4.49E-03	up	0.82
hsa-miR-204-5p|0|0	TTCCCTTTGTCATCCTATGCCT	canonical	1.91E-02	up	1.95
hsa-miR-204-5p|0|0(+2U)	TTCCCTTTGTCATCCTATGCCTTT	3’ non-templated addition	2.46E-02	up	0.87
hsa-miR-204-5p|0|−1	TTCCCTTTGTCATCCTATGCC	end-site	1.40E-02	up	2.09
hsa-miR-204-5p|0|−2	TTCCCTTTGTCATCCTATGC	end-site	1.60E-02	up	1.76
hsa-miR-214-5p|+1|0	GCCTGTCTACACTTGCTGTGC	start-site	2.59E-02	up	0.96
hsa-miR-296-3p|0|−2	GAGGGTTGGGTGGAGGCTCT	end-site	3.53E-05	up	0.98
hsa-miR-29a-3p|0|−4(+2U)	TAGCACCATCTGAAATCGTT	mixed: end-site + 3’ non-templated addition	4.15E-03	up	0.85
hsa-miR-30c-2-3p|0|−2	CTGGGAGAAGGCTGTTTACT	end-site	1.39E-02	up	0.72
hsa-miR-330-5p|0|−1	TCTCTGGGCCTGTGTCTTAGG	end-site	2.54E-02	up	0.78
hsa-miR-342-3p|0|0(+1U)	TCTCACACAGAAATCGCACCCGTT	3’ non-templated addition	4.49E-03	up	0.70
hsa-miR-342-5p|+1|+2	GGGGTGCTATCTGTGATTGAGG	mixed: start-site + end-site	1.09E-02	up	0.61
hsa-miR-381-3p|0|0	TATACAAGGGCAAGCTCTCTGT	canonical	2.52E-02	up	0.94
hsa-miR-409-5p|0|0	AGGTTACCCGAGCAACTTTGCAT	canonical	4.97E-02	up	0.93
hsa-miR-423-5p|0|−2	TGAGGGGCAGAGAGCGAGACT	end-site	2.54E-02	up	1.06
hsa-miR-432-5p|0|−1(+1U)	TCTTGGAGTAGGTCATTGGGTGT	mixed: end-site + 3’ non-templated addition	4.64E-02	up	1.00
hsa-miR-485-3p|0|0	GTCATACACGGCTCTCCTCTCT	canonical	3.05E-02	up	0.90
hsa-miR-493-3p|0|−1	TGAAGGTCTACTGTGTGCCAG	end-site	9.25E-03	up	0.92
hsa-miR-495-3p|0|0	AAACAAACATGGTGCACTTCTT	canonical	8.45E-03	up	0.89
hsa-miR-518a-3p|0|0	GAAAGCGCTTCCCTTTGCTGGA	canonical	1.91E-02	up	2.55
hsa-miR-519a-5p|−1|−1	ACTCTAGAGGGAAGCGCTTTCT	shifted	2.44E-04	up	3.59
hsa-miR-625-3p|+1|−1(+1U)	ACTATAGAACTTTCCCCCTCT	mixed: start-site + end-site + 3’ non-templated addition	3.90E-02	up	0.85
hsa-miR-671-3p|0|0	TCCGGTTCTCAGGGCTCCACC	canonical	3.95E-02	up	0.84
hsa-miR-6892-5p|0|−1	GTAAGGGACCGGAGAGTAGG	end-site	2.44E-04	up	0.89
hsa-miR-758-5p|+2|+1	TGGTTGACCAGAGAGCACACG	mixed: start-site + end-site	1.90E-03	up	0.91
hsa-miR-92a-3p|0|+1(+1U)	TATTGCACTTGTCCCGGCCTGTGT	mixed: end-site + 3’ non-templated addition	2.14E-02	down	−1.31
hsa-miR-937-3p|0|−2	ATCCGCGCTCTGACTCTCTG	end-site	6.44E-03	up	1.09
hsa-miR-942-5p|0|−2	TCTTCTCTGTTTTGGCCATG	end-site	7.63E-07	up	0.93
**Comparison between BRAF V600E and BRAF WT samples**					
Name	Sequence	Type	p (Corr) ([V600E vs [WT])	Regulation FC ([V600E vs. [WT])	FC ([V600E vs. [WT])
hsa-let-7b-5p|0|−1	TGAGGTAGTAGGTTGTGTGGT	end-site	0.0289	up	2.13
hsa-let-7b-5p|0|−2	TGAGGTAGTAGGTTGTGTGG	end-site	0.0262	up	2.32
hsa-miR-100-5p|+1|−1	ACCCGTAGATCCGAACTTGT	mixed: start-site + end-site	0.0234	up	4.35
hsa-miR-100-5p|0|0	AACCCGTAGATCCGAACTTGTG	canonical	0.0262	up	3.62
hsa-miR-100-5p|0|−1	AACCCGTAGATCCGAACTTGT	end-site	0.0089	up	4.59
hsa-miR-125b-5p|0|0	TCCCTGAGACCCTAACTTGTGA	canonical	0.0323	up	2.70
hsa-miR-125b-5p|0|−1	TCCCTGAGACCCTAACTTGTG	end-site	0.0262	up	3.02
hsa-miR-146b-3p|0|0	GCCCTGTGGACTCAGTTCTGGT	canonical	0.0146	up	2.33
hsa-miR-181c-5p|0|0	AACATTCAACCTGTCGGTGAGT	canonical	0.0262	up	2.22
hsa-miR-204-5p|0|0	TTCCCTTTGTCATCCTATGCCT	canonical	0.0289	up	4.63
hsa-miR-181a-2-3p|0|+1	ACCACTGACCGTTGACTGTACCT	end-site	0.0241	down	−2.91
hsa-miR-181a-2-3p|−1|−1	AACCACTGACCGTTGACTGTAC	shifted	0.0483	down	−2.29
**Comparison between BRAF V600E-V600M and BRAF V600E samples**					
Name	Sequence	Type	*p* (Corr) ([V600E | V600M] Vs. [V600E])	Regulation ([V600E | V600M] Vs. [V600E])	FC ([V600E | V600M] Vs. [V600E])
hsa-let-7b-3p|0|−1(+1U)	CTATACAACCTACTGCCTTCCT	mixed: end-site + 3’ non-template addition	0.03969	down	−2.30
hsa-miR-100-5p|0|0(+1U)	AACCCGTAGATCCGAACTTGTGT	3’ non-template addition	0.03969	down	−4.14
hsa-miR-125b-5p|0|0	TCCCTGAGACCCTAACTTGTGA	canonical	0.03969	down	−2.77
hsa-miR-221-3p|0|−1	AGCTACATTGTCTGCTGGGTTT	end-site	0.03969	down	−2.29
hsa-let-7a-5p|+1|−2	GAGGTAGTAGGTTGTATAG	mixed: start-site + end-site	0.03969	up	2.14
hsa-miR-1247-5p|0|−1	ACCCGTCCCGTTCGTCCCCGG	end-site	0.03969	up	4.07
hsa-miR-219a-1-3p|0|0	AGAGTTGAGTCTGGACGTCCCG	canonical	0.03969	up	1.75

**Table 8:** List of mature microRNAs associated with NRAS mutation in TCGA SKCM samples.

Comparison between Mutated NRAS and Wild Type (WT) NRAS					
Name	Sequence	Type	*p* (Corr)(Mutated NRAS Vs. Wild Type NRAS)	Regulation(Mutated NRAS Vs. Wild Type NRAS)	Log FC(Mutated NRAS Vs. Wild Type NRAS)
hsa-miR-17-3p|+1|0	CTGCAGTGAAGGCACTTGTAG	start-site	8.11E-02	up	0.92
hsa-miR-17-3p|0|0	ACTGCAGTGAAGGCACTTGTAG	canonical	2.11E-02	up	0.85
hsa-miR-19b-3p|0|−1	TGTGCAAATCCATGCAAAACTG	end-site	8.11E-02	up	0.79
hsa-miR-20a-5p|0|−2	TAAAGTGCTTATAGTGCAGGT	end-site	7.26E-02	up	0.85
hsa-miR-3614-5p|0|−1	CCACTTGGATCTGAAGGCTGCC	end-site	8.11E-02	up	0.85
hsa-miR-509-3p|+4|+1	TGGTACGTCTGTGGGTAGA	mixed: start-site + end-site	4.66E-02	down	−1.10

## Data Availability

The small RNA sequencing raw data (FASTQ format) are available at the European Nucleotide Archive (ENA) with the following accession number: PRJEB35819.

## Supplementary Materials

The following are available online at https://www.mdpi.com/article/10.3390/ncrna7040063/s1. Table S1: List of mature microRNAs detected in the small-RNA sequencing of 23 samples. Table S2: List of isomiRs that are most expressed in 20 early-stage melanoma samples. Table S3: List of isomiR/canonical miRNA ratios in 20 early-stage melanoma samples. Table S4: List of mature microRNAs identified in TCGA SKCM data. Table S5: List of isomiR/canonical miRNA ratios in 63 fresh-frozen melanoma samples from TCGA. Table S6: List of mature microRNAs differentially expressed in melanoma metastasis compared to primary melanomas from TCGA SKCM dataset. Table S7: List of TCGA melanoma samples with the mutation status of BRAF, NRAS and NF1. Figure S1: List of 332 mature microRNAs differently expressed in melanoma metastasis compared to primary melanoma. Figure S2: Opposite expression trend of mature microRNAs in melanoma metastasis and primary melanoma.
