# Supplementary material for: MicroRNA Isoforms Contribution to Melanoma Pathogenesis
Source: Noncoding RNA. 2021 Sep 27;7(4):63. doi: 10.3390/ncrna7040063 (PMC8544706; doi:10.3390/ncrna7040063)
Supplement: Supplementary file 1 [file ncrna-07-00063-s001.zip › ncrna-1316551-Supplementary Materials.pdf]

non-coding RNA

MicroRNA Isoforms Contribution to Melanoma Pathogenesis

Supplementary Materials

Supplementary Table S1: List of mature microRNAs detected in the small-RNA sequencing of 23 samples.

Supplementary Table S2: List of isomiRs that are most expressed in 20 early-stage melanoma samples.

Supplementary Table S3: List of isomiR/canonical miRNA ratios in 20 early-stage melanoma samples.

Supplementary Table S4: List of mature microRNAs identified in TCGA SKCM data.

Supplementary Table S5: List of isomiR/canonical miRNA ratios in 63 fresh-frozen melanoma samples from TCGA.

Supplementary Table S6: List of mature microRNAs differentially expressed in melanoma metastasis compared to primary melanomas from TCGA SKCM dataset.

Supplementary Table S7: List of TCGA melanoma samples with the mutation status of BRAF, NRAS and NF1.

Supplementary Figure S1: List of 332 mature microRNAs differently expressed in melanoma metastasis compared to primary melanoma. Interleaved bar charts showed average log fold change of 211 upregulated (in red) (**A**) and 121 downregulated (in blue) (**B**) mature microRNAs in melanoma metastasis ($n = 260$) compared to primary melanoma ($n = 63$). Comparison was performed using the unpaired t-test (FDR 5% with Benjamini-Hochberg correction).

Supplementary Figure S2: Opposite expression trend of mature microRNAs in melanoma metastasis and primary melanoma. Before-after blot of differently expressed mature microRNAs showing a different expression trend for at least two isoforms of the same canonical miRNA. The tables report name, normalized expression in melanoma metastasis (average of 260 samples) and primary melanoma (average of 63 samples) and the p value of the comparison.
